# Impact of frailty on survival and readmission in patients with gastric cancer undergoing gastrectomy: A meta-analysis

**DOI:** 10.3389/fonc.2022.972287

**Published:** 2022-10-31

**Authors:** Xiaoyan Wang, Yimeng Sun, Pei Wang, Yu Jie, Guodong Liu, Dandan Gong, Yu Fan

**Affiliations:** ^1^ Department of Gastroenterology, The Suqian Clinical College of Xuzhou Medical University, Suqian, Jiangsu, China; ^2^ Cancer Institute, The Affiliated People’s Hospital, Jiangsu University, Zhenjiang, Jiangsu, China; ^3^ Department of General Surgery, The Suqian Clinical College of Xuzhou Medical University, Suqian, Jiangsu, China

**Keywords:** frailty, gastric cancer, gastrectomy, overall survival, disease-specific survival, meta-analysis

## Abstract

**Background:**

Frailty as a common geriatric syndrome can affect the clinical outcomes in patients with gastric cancer. However, the impact of frailty on survival and readmission patients with gastric cancer has not been well-characterised.

**Objectives:**

To investigate the impact of frailty on survival and readmission in patients with gastric cancer undergoing gastrectomy by conducting a meta-analysis.

**Methods:**

Eligible studies were identified by searching the PubMed, Web of Science, Cochrane Library, and Embase databases until 2 September 2022. Observational studies that evaluated the value of frailty in predicting adverse outcomes in gastric cancer patients undergoing gastrectomy were included. The outcomes of interest were overall survival, disease-specific survival (death from gastric cancer), and readmission. Adjusted hazard ratios (HR) with 95% confidence intervals (CI) were pooled to calculate the association of frailty with adverse outcomes.

**Results:**

Eight studies reported on nine articles with 2,792 patients with gastric cancer were included. A fixed-effect meta-analysis indicated that frailty was associated with a reduced in-hospital overall survival (HR 2.08; 95% CI 1.46–2.95), long-term overall survival (HR 1.84; 95% CI 1.37–2.47), and disease-specific survival (HR 1.94; 95% CI 1.34–2.83). In addition, frailty was associated with increased risk of readmission within 1 year (HR 3.63; 95% CI 1.87–7.06).

**Conclusions:**

Frailty was associated with a reduced overall survival and disease-specific survival and an increased risk of readmission in patients with gastric cancer undergoing gastrectomy. Frail status may play an important role in the risk stratification of gastric cancer after gastrectomy.

## Introduction

Gastric cancer is the fifth most common malignancy that is responsible for more than 1 million new cases annually worldwide (1). Gastrectomy is the main curative treatment for gastric cancer (2). Despite the advancement in surgical treatment, the mortality rate from gastric cancer remains substantially high (3). Gastric cancer frequently occurs in elderly people. The incidence rates for gastric cancer increase with advanced age (4).Therefore, comprehensive geriatric assessment may help in improving the risk classification of gastric cancer.

Frailty is a geriatric syndrome characterised by a decline in physiological reserve and functioning across multiple-organ systems (5, 6). This geriatric syndrome increases with aging (7). Frailty is a promising predictor for adverse health outcomes in older patients with cancer (8, 9). Frailty is associated with increased risk of postoperative morbidity and mortality among malignant or benign diseases in the stomach among patients who underwent gastrectomy (10). An early systematic review only described the association between frailty and adverse outcomes in patients with gastric cancer undergoing gastrectomy (11). Subsequently, several new articles (12–17) that investigated the predictive utility of frailty in patients with gastric cancer have pursued a meta-analysis that applies accumulating clinical evidence. Nevertheless, the value of the frailty in predicting overall survival remains conflicting in patients with gastric cancer (17, 18).

To address these knowledge gaps, we performed this more focused meta-analysis to examine the impact of frailty on survival and readmission in patients with gastric cancer undergoing gastrectomy.

## Materials and methods

### Search strategy

This study was conducted following the Preferred Reporting Items for Systematic Reviews and Meta-Analyses guidelines (19). Two independent authors comprehensively searched the studies indexed in PubMed, Web of Science, Cochrane Library, and Embase databases until 2 September 2022. The following combined keywords were used for literature search: “frailty” OR “frail” AND “gastric cancer” OR “stomach cancer” AND “gastrectomy” OR “gastric surgery” (**Supplemental Text S1**). A manual search of reference lists of included studies and pertinent reviews were performed to identify any additional studies.

### Study selection

Studies that met the following criteria were included: 1) population: patients with gastric cancer undergoing gastrectomy; 2) predictor: frailty assessed using a valid tool before gastric surgery; 3) comparison: individuals with frailty versus those without; 4) outcome measures: overall survival, disease-specific survival (defined by death from the gastric cancer), or readmission; 5) study design: retrospective or prospective cohort studies published in peer-reviewed journals; and 6) reported the risk estimate associated frailty in multivariate regression analyses. For multiple articles from the same population, only articles with the most comprehensive data were included. The exclusion criteria were as follows: 1) not restricted in patients with gastric cancer; 2) outcome measures were not of interest; and 3) conference abstract, letter, or unpublished studies.

### Data extraction and quality assessment

Two authors independently extracted the last name of the first author, year of publication, country, study design, sample size, age of patients, gender distribution, surgical type, assessment of frail tool, outcome measures, follow-up duration, fully adjusted risk summary associated with frailty, and variable adjustment from eligible studies. The Newcastle–Ottawa Scale (NOS) for cohort studies was used to assess the risk of bias of the included studies (20). A total score of seven points or over indicates a low risk of bias (high-quality). Any disagreements were settled through a discussion between two authors until consensus was reached. When information was not reported in the original article, we contacted the corresponding author by e-mail for the missing data.

### Data analysis

Stata software 12.0 (Stata, College Station, TX, USA) was used for all meta-analyses.

If the relative risk of an outcome is reported as odds ratio (OR), we calculated the hazard ratio (HR) from the OR by using the following formula: HR = OR/[(1-Prevalence _non-exposed group_) + (Prevalence _non-exposed group_× OR)]. The impact of frailty on adverse outcomes was expressed by pooling the fully adjusted HR with 95% confidence intervals (CI) from individual studies. Between-study heterogeneity was investigated using the I^2^ statistics and Cochrane Q test. A fixed-effect model was selected in the absence of significant heterogeneity (*p*-value >0.10 of the Cochrane Q test or I^2^ statistic value <50%). Sensitivity analysis was performed by sequentially removing an individual study for each turn. Begg’s test (21) and Egger’s test (22) were scheduled to investigate publication bias when the outcomes were reported in more than 10 studies. The overall certainty of evidence was summarised using the GRADE framework.

## Results

### Search results and study characteristics

**Figure 1:**
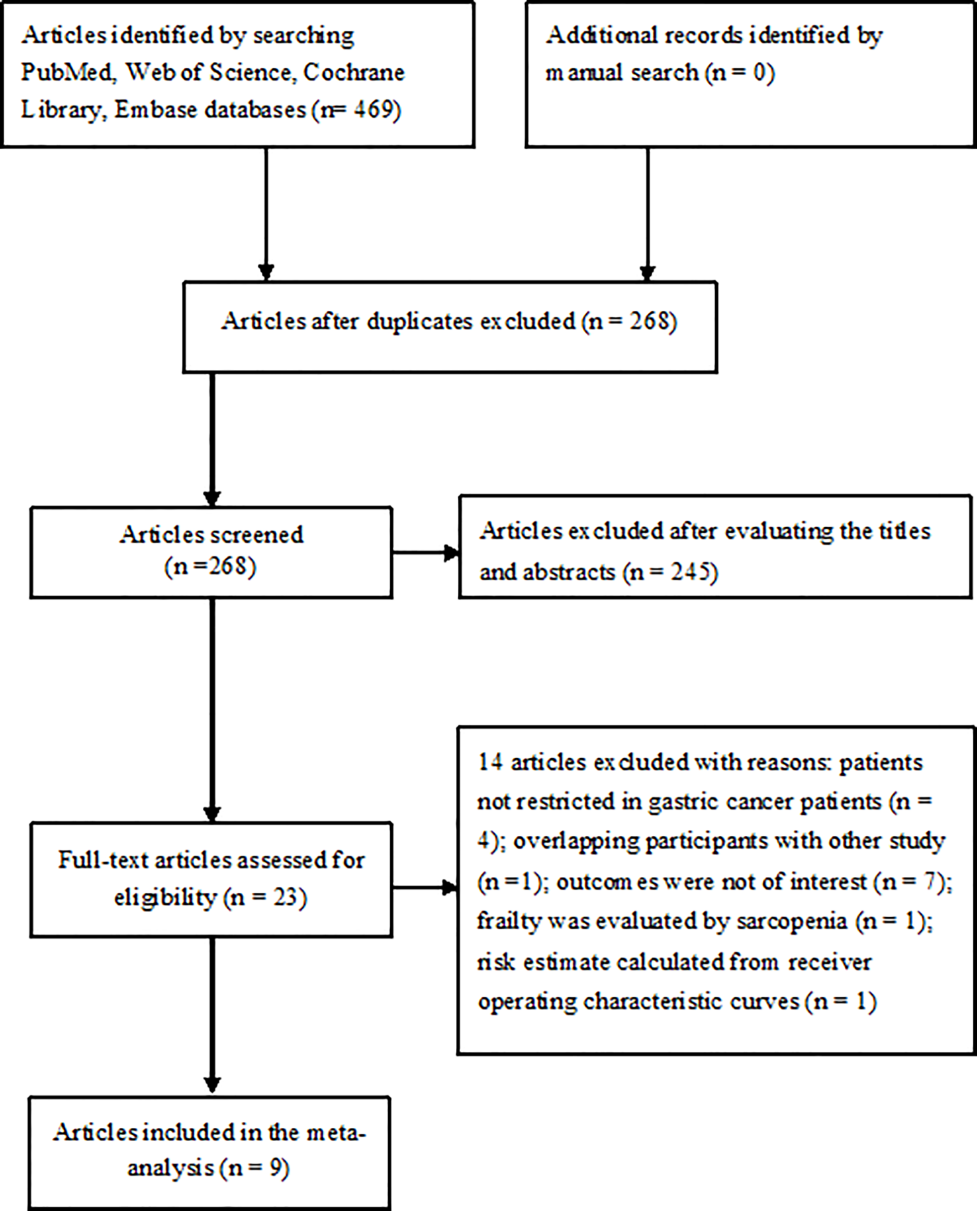
Flowchart showing the process of study selection.

The literature search yielded 469 potentially relevant articles, of which 201 articles were directly excluded as duplicate. After evaluating the titles and abstracts, 23 articles were retrieved for full-text evaluation. Two articles (12, 23) were obtained from the same population and reported the different clinical outcomes. Finally, eight studies reported on nine articles (12, 13, 15–18, 23–25) were included in the meta-analysis (**Figure 1**).

The major characteristic of the included studies is summarised in **Table 1**. These studies were published from 2014 to 2022 and conducted in South Korea (12, 23), Republic of Korea (17), Japan (15, 16), The Netherlands (18), USA (25), and China (13). Two articles (13, 17) adopted a prospective design, while the others adopted a retrospective design. The sample sizes of individual studies ranged from 96 to 1,173, with a total of 2,792 patients with gastric cancer. The Clinical Frailty Scale (15, 16), Modified Frailty Index (13, 24), Groningen Frailty Indicator (18), Study of Osteoporotic Fractures Index (12, 23), Johns Hopkins Adjusted Clinical Groups Frailty criteria (25), and Multidimensional Frailty Score (17) tools were used to assess frailty. The detailed components used to assess frailty are summarised in **Table 2**. According to the frailty tool used, the prevalence of frailty varied from 14.8% to 72.3%. The duration of follow-up was up to 60 months. Based on the NOS criteria, all the included articles were grouped to have high-quality (**Supplemental Table S1**).

**Table 1:** Main characteristics of the included studies.

Author/year	Region	Study design	Sample size (% men)	Age (years)	Therapy	Follow-up	Frailty criteria/Prevalence (%)	Outcomes/HR (95% CI)	Adjusted for variables
Tegels 2014 (18)	Netherlands	R	180 (58.9)	69.8 (37–88)	Gastrectomy	Hospital	Groningen Frailty Indicator (≥3); 16.7%	Overall survival 3.43 (1.11–10.64) #	Age, ASA, neoadjuvant chemotherapy, type of surgery, tumour stage
Choe 2017 (12)	South Korea	R	233 (58.4)	72.1 ± 4.6	Gastrectomy	12 months	SOF Index (≥2 items); 14.8%	Readmission 4.28 (1.62–11.32) #	Age, sex, performance status, histological type, stage
Lu 2017 (13)	China	P	165 (80)	80-93	Gastrectomy	30.8 months	Modified Frailty Index (>2 items); 32.7%	Overall survival 1.61 (1.05–2.47) DSS 1.61 (1.03–2.51)	Tumour size, tumour stage, ASA, NLR, PLR, prognostic nutritional index
Tanaka 2019 (15)	Japan	R	96 (72.9)	82 (80–92)	Laparoscopic gastrectomy	60 months	Clinical Frailty Scale (≥5); 17.7%	Overall survival 1.97 (1.21–3.20) # DSS 2.90(1.15–7.30) #	Age, sex, BMI, stage, performance status, haemoglobin, type of resection, lymphadenectomy, morbidity, ASA, Charlson Comorbidity Index, prognostic nutritional index
Misawa 2020 (16)	Japan	R	142 (64.1)	83.7 ± 3.0	ESD	48 months	Clinical Frailty Scale (≥4); 28.9%	Overall survival 2.47 (1.02–5.98)	Performance status, Charlson comorbidity index, Onodera prognostic nutritional index
Kim 2020 (17)	Republic of Korea	P	289 (63.3)	77.3 (66-94)	Gastrectomy	12 months	Multidimensional Frailty Score (>5); 38.4%	Overall survival 2.06 (0.62–6.79) #	Age, sex, pathological stage of cancer, type of gastrectomy
Osaki 2021 (24)	Japan	R	516 (73.1)	Not provided	Gastrectomy	12 months	Modified Frailty Index-11 (≥0.14); 72.3%	Readmission 3.15 (1.27–7.83)	Age, sex, BMI, stage, type of approach, prognostic nutritional index, chemotherapy, complication, nonhome discharge
Jeong 2022 (23)	South Korea	R	231 (60.6)	72.0 ± 4.9	Gastrectomy	48 months	SOF Index (≥2 components); 15.2%	DSS 3.33 (1.16–9.56)	TNM stage, type of approach, total gastrectomy, lymph node dissection
Lee 2022 (25)	USA	R	1171 (56.7)	Mean 68.3	Gastrectomy	Hospital	Johns Hopkins ACG Frailty criteria (≥1 item); 22.15%	Overall survival 1.97 (1.36–2.84) #	Multivariate analysis

HR, hazard ratio; CI, confidence intervals; R, retrospective; P, prospective; NP, not provided; DSS, disease-specific survival; ESD, endoscopic submucosal dissection; SOF, Study of Osteoporotic Fractures; ACG, Adjusted Clinical Groups; ASA, American Society of Anesthesiologists; NLR, neutrophil lymphocyte ratio; PLR, platelet lymphocyte ratio; BMI, body mass index; DM, diabetes mellitus; ASA, American Society of Anesthesiologists. #Results calculating from the reported odds ratio.

**Table 2:** Components included in different frailty tools.

Modified Frailty Index-11	Modified Frailty Index	Clinical Frailty Scale	Groningen Frailty Indicator	Study of Osteoporotic Fractures Index	Johns Hopkins ACG Frailty criteria	Multidimensional Frailty Score
Cognitive impairment	Albumin <3.4 g/dl	Physical function	Mobility	Involuntary weight loss	Dementia	Malignant disease
Functional dependent	Haematocrit <35%	ADLs	Physical fitness	Rise from a chair	Housing needs	Charlson Comorbidity Index
Myocardial infarction	Creatinine >2 mg/dl	Comorbidity	Vision	Reduced energy level	Difficult ambulation	Albumin
CAD			Hearing		Weight loss	ADLs
Congestive heart failure			Morbidity		Vision impairment	Lawton and Brody Index
Stroke			Cognition		Frequent falls	Dementia
Diabetes			Psychosocial		Urinary incontinence	Delirium
Hypertension			Nourishment		Faecal incontinence	MNA
COPD					Pressure ulcerations	Midarm circumference
Delirium					Feeding difficulty	
					Malnutrition	

COPD, chronic obstructive pulmonary disease; CAD, coronary artery disease; ADLs, activities of daily livings; ACG, Adjusted Clinical Groups; MNA, Mini Nutritional Assessment.

### Overall survival

**Figure 2:**
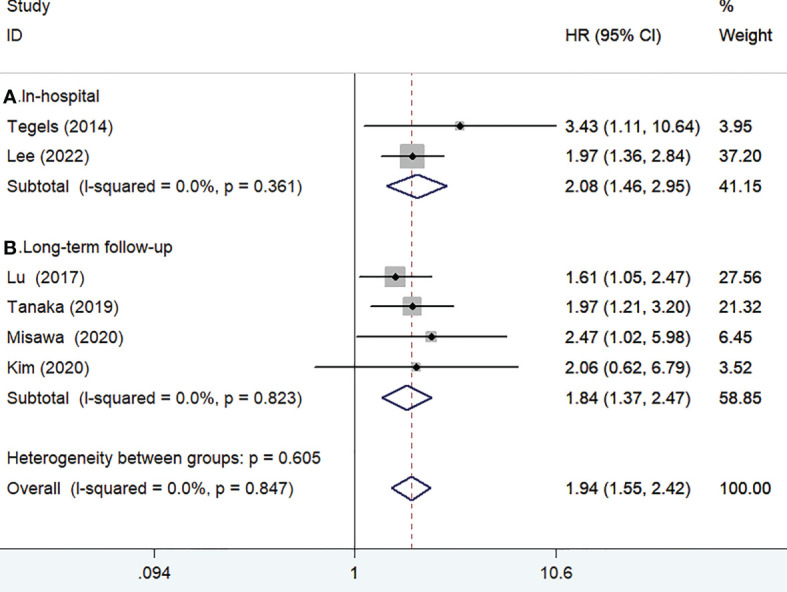
Forest plots showing the pooled HR with 95% CI of in-hospital **(A)** and long-term overall survival for the frail patients.

Six studies (13, 15–18, 25) reported the impact of frailty on overall survival. As shown in **Figure 2**, no heterogeneity was observed across studies (I^2^ = 0%, *p* = 0.847). A fixed-effect model meta-analysis showed that frailty was associated with a reduced overall survival (HR 1.94; 95% CI 1.55–2.42). Subgroup analysis on the duration of follow-up showed that frailty was associated with a reduced in-hospital overall survival (HR 2.08; 95% CI 1.46–2.95; **Figure 2A**) and long-term overall survival (HR 1.84; 95% CI 1.37–2.47; **Figure 2B**). After removing the most influential study (Lu 2017), the pooled HR of long-term overall survival was 1.84 (95% CI 1.37–2.47). The results of the leave-out one study sensitivity analysis showed that the pooled HR of long-term overall survival varied from 1.77 to 2.07 (all *p*-value <0.05). Begg’s test (n = 0.452) and Egger’s test (n = 0.142) suggested no evidence of publication bias.

### Disease-specific survival

**Figure 3:**
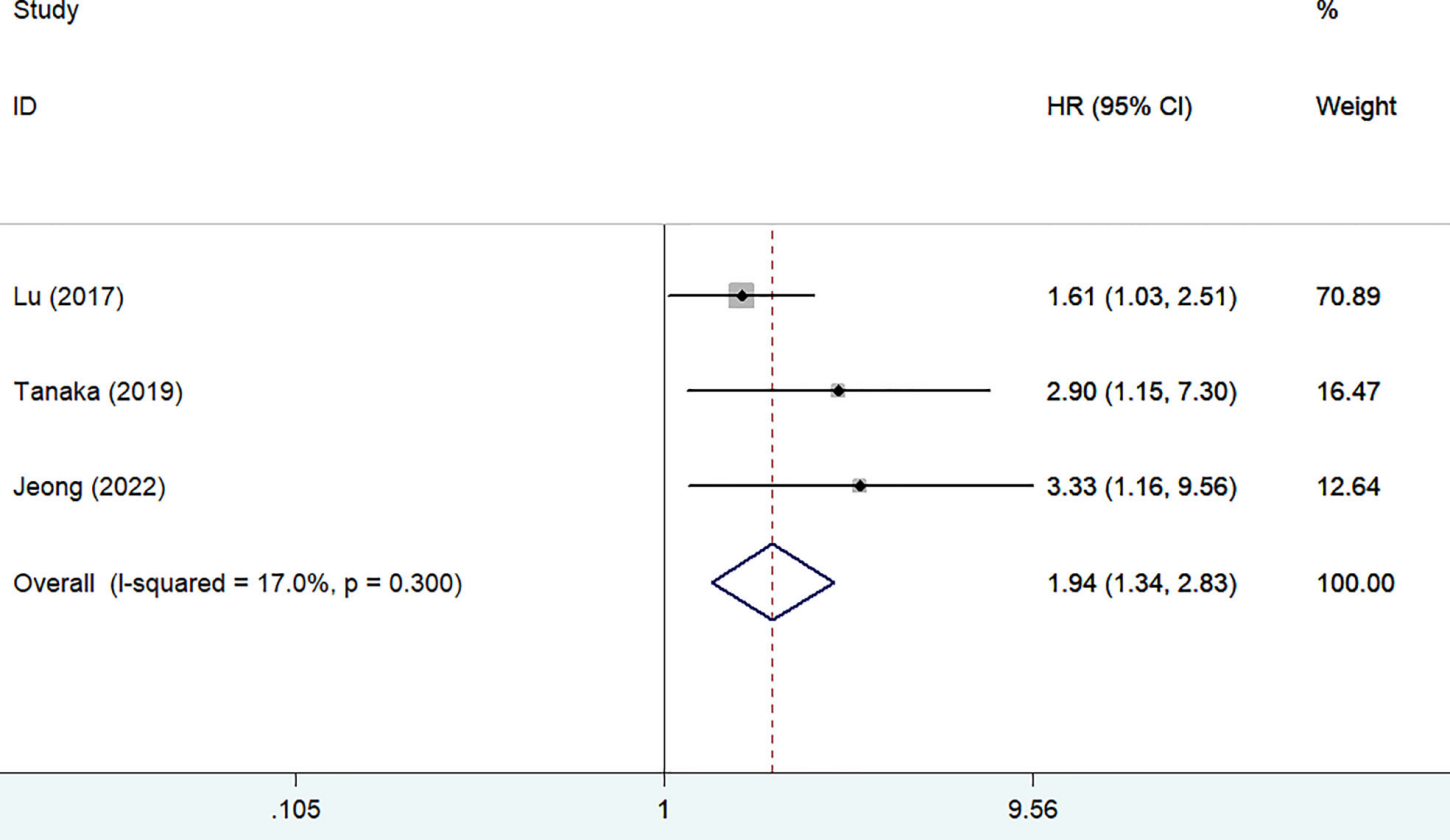
Forest plots showing the pooled HR with 95% CI of disease-specific survival for the frail patients.

**Figure 4:**
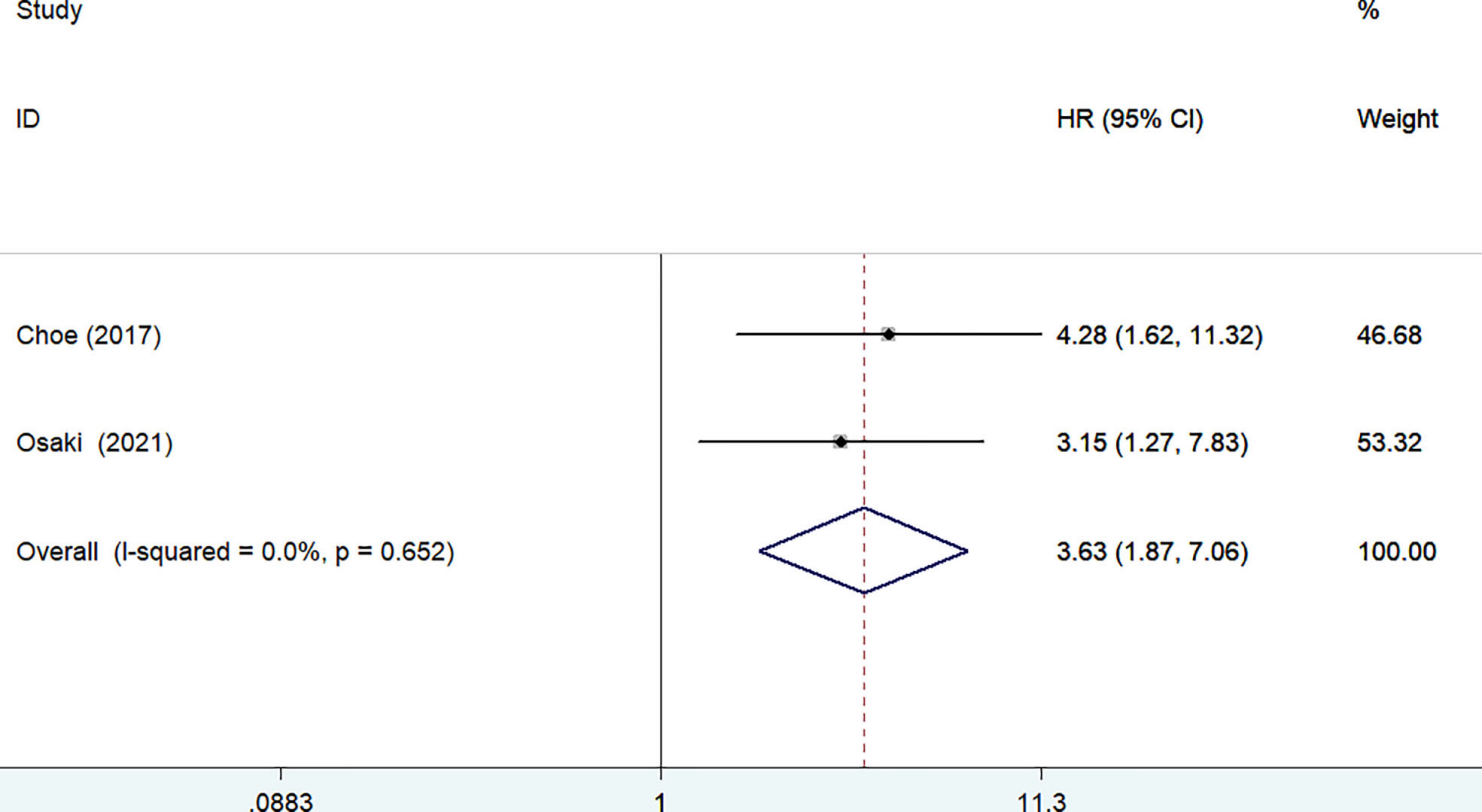
Forest plots showing the pooled HR with 95% CI of readmission for the frail patients.

Three studies (13, 15, 23) reported the impact of frailty on disease-specific survival. As shown in **Figure 3**, no significant heterogeneity was observed between studies (I^2^ = 17.0%, *p* = 0.300). A fixed-effect model meta-analysis showed that frailty was associated with a reduced disease-specific survival (HR 1.94; 95% CI 1.34–2.83). After removing the most influential study (Lu 2017), the pooled HR was 3.08 (95% CI 1.54–6.17) for disease-specific survival. The results of the leave-out one study sensitivity analysis suggested that the pooled HR of disease-specific survival ranged from 1.80 to 3.08 (all *p*-values <0.05).Two studies (12, 24) reported the impact of frailty on readmission within 1 year. As shown in **Figure 4**, no heterogeneity (I^2^ = 0%, *p* = 0.652) was observed between studies. A fixed-effect model meta-analysis showed that frailty was associated with an increased risk of readmission (HR 3.63; 95% CI 1.87–7.06).

### GRADE certainty of evidence

The overall certainty of evidence for overall survival was high, and that for disease-specific survival/readmission was low (**Supplemental Table S2**). The downgrading evidence quality for disease-specific survival/readmission can be attributed to imprecision (small number of participants) and unclear risk of publication bias.

## Discussion

The current meta-analysis focused on the impact of preoperative frailty on adverse clinical outcomes in patients with gastric cancer undergoing gastrectomy. The meta-analysis results confirmed that frailty was associated with a reduced overall survival and disease-specific survival among these patients. Patients with gastric cancer undergoing gastrectomy having frailty conferred a 2.08-fold and 84% poor in-hospital and long-term follow-up overall survival. Frailty was also associated with 94% poor disease-specific survival. In addition, patients with gastric cancer having frailty had a 3.63-fold higher risk of readmission within 1 year follow-up. Therefore, frailty may provide important prognostic information in patients with gastric cancer.

Apart from the abovementioned outcomes, frailty, as defined by the Modified Frailty Index, was an independent predictor of non-home discharge (26) and postoperative pulmonary infection (27) in elderly patients with gastric cancer who underwent gastrectomy. In patients with gastric cancer above the age of 80 years, frailty independently predicted the recurrence-free survival (13). Frailty was associated with a higher risk of postoperative complications in patients with gastric cancer after surgery (14, 18). Moreover, frailty, as defined by the Johns Hopkins Adjusted Clinical Groups criteria, conferred a 40% higher risk of length of the hospital stay in patients undergoing gastrectomy for gastric cancer (25). These findings further supported frailty as an important prognostic indicator in patients with gastric cancer undergoing gastrectomy.

Seven frailty tools were used in the current meta-analysis. However, the frailty tools with the superior predictive value cannot be compared because of the small number of studies included. Future prospective studies are required to determine which frailty tool can accurately predict adverse outcomes in patients with gastric cancer. No frailty tool has been well accepted for use among patients with gastric cancer. Nutritional status is an important aspect in patients with gastric cancer because their intake is usually restricted by mechanical obstruction. Malnutrition estimated by a low prognostic nutritional index (28) or higher controlling nutritional status score (29) is associated with a poor prognosis following gastrectomy for gastric cancer. Therefore, a combination of self-reported items and nutritional status should be considered to estimate the frailty in a clinical setting for patients with gastric cancer. A valid frailty tool needs to be developed from a cancer-specific geriatric assessment in older adults with gastric cancer.

Depending on the tool of frailty, the prevalence of frailty varied from 14.8% to 72.3% among patients with gastric cancer before gastrectomy. Our meta-analysis highlights the incorporation of frailty assessment before surgery can improve risk stratification in patients with gastric cancer. A thorough assessment of frail status can help oncologists to identify patients with gastric cancer at risk for mortality, postoperative complications, and other adverse outcomes. Frailty may also affect the clinical decision-making and individualised treatment strategies. Early intervention of frailty may help to improve survival and reduce admission in patients with gastric cancer.

The management of patients with gastric cancer faces many challenges during the COVID-19 pandemic. The COVID-19 outbreak has affected the diagnosis and treatment of many patients with gastric cancer (30, 31). Delayed diagnosis and extended waiting times for surgery could advance the cancer stage and deteriorate the survival outcome (32). Therefore, strategies should be developed for gastric cancer patients with frailty during the COVID-19 pandemic.

The strengths of our meta-analysis included the inclusion of high-quality studies and low heterogeneity between these studies. However, it has several limitations. First, majority of the included studies were of retrospective designs, which carried an inherent selection bias. Second, various methods of frailty assessment across studies remarkably limit the results. An inadequate definition of frailty may have affected the prognostic utility of frailty. Third, subgroup analysis was not performed according to the gender, type of surgery (open procedures or laparoscopy), or gastrectomy (partial or total) because of insufficient such data. Future studies should further investigate whether the prognostic value of frailty is affected by these factors. Only two studies were included in the analysis of readmission. Therefore, this outcome should be interpreted with caution because meta-analysis that involves two studies may result in biased information. Fourth, the result of the publication bias for overall survival may be potentially unreliable because the number of studies was less than the recommended arbitrary minimum number of 10 (33). Moreover, a publication test for disease-specific survival and readmission outcomes was not carried out because of the small number of studies included. Fifth, surgical procedure (17), pre-morbid functional status or cancer stage (34), and number and site of lymph node invasions (35) are associated with the prognosis of patients with gastric cancer. Lack of adjustment in these factors could have an effect on the prognostic utility of frailty. Finally, our meta-analysis could not determine the frailty tool with the best predictive value in patients with gastric cancer undergoing gastrectomy. Future well-designed studies are required to directly compare the predictive utility of different frailty tools in this population.

## Conclusions

Frailty was associated with a reduced overall survival and disease-specific survival and increased risk of readmission in patients with gastric cancer undergoing gastrectomy. Frail status may play an important role in risk stratification of gastric cancer after gastrectomy.

## Data availability statement

The original contributions presented in the study are included in the article/**Supplementary Material**. Further inquiries can be directed to the corresponding authors.

## Author contributions

YF and DG contributed to study design and guaranteed the integrity of study. XW, YS, and PW searched the literature, extracted the data, assessed the study quality, and conducted the statistical analysis. GL drafted the manuscript. XW revised/edited the manuscript. All the authors have read and approved the final version of manuscript.

## Funding

This work is supported by the Suqian Science and Technology Support Project Fund (K202014), the Zhenjiang Key Research and Development Fund (SH2021038), and the Jiangsu 333 Talent Fund (WSW205, WSW236).

## Conflict of interest

The authors declare that the research was conducted in the absence of any commercial or financial relationships that could be construed as a potential conflict of interest.

## Publisher’s note

All claims expressed in this article are solely those of the authors and do not necessarily represent those of their affiliated organizations, or those of the publisher, the editors and the reviewers. Any product that may be evaluated in this article, or claim that may be made by its manufacturer, is not guaranteed or endorsed by the publisher.
