# Supplementary material for: Impact of frailty on survival and readmission in patients with gastric cancer undergoing gastrectomy: A meta-analysis
Source: Front Oncol. 2022 Oct 31;12:972287. doi: 10.3389/fonc.2022.972287 (PMC9659614; doi:10.3389/fonc.2022.972287)
Supplement: Supplementary file 1 [file DataSheet_1.docx]

**Supplemental Text S1 – Search strategy**

**Databases---PubMed, Embase, Web of Science Strategy, and Cochrane Library**

**Limits:** Publications until September 2, 2022

**1. PubMed databases:**

| **PubMed Search** | Query | Items found |
| --- | --- | --- |
| #1 | Search: **(frailty) OR (frail)** | 37,319 |
| #2 | **Search: (((gastric cancer) OR (stomach cancer)) OR (gastrectomy)) OR (gastric surgery)** | 275,007 |
| #3 | Search #1 or #2 | 167 |

**Search detail in PubMed database**

("frailty"[MeSH Terms] OR "frailty"[All Fields] OR "frailties"[All Fields] OR ("frail"[All Fields] OR "frails"[All Fields] OR "frailty"[MeSH Terms] OR "frailty"[All Fields] OR "frailness"[All Fields])) AND ("stomach neoplasms"[MeSH Terms] OR ("stomach"[All Fields] AND "neoplasms"[All Fields]) OR "stomach neoplasms"[All Fields] OR ("gastric"[All Fields] AND "cancer"[All Fields]) OR "gastric cancer"[All Fields] OR ("stomach neoplasms"[MeSH Terms] OR ("stomach"[All Fields] AND "neoplasms"[All Fields]) OR "stomach neoplasms"[All Fields] OR ("stomach"[All Fields] AND "cancer"[All Fields]) OR "stomach cancer"[All Fields]) OR ("gastrectomy"[MeSH Terms] OR "gastrectomy"[All Fields] OR "gastrectomies"[All Fields]) OR (("gastrics"[All Fields] OR "stomach"[MeSH Terms] OR "stomach"[All Fields] OR "gastric"[All Fields]) AND ("surgery"[MeSH Subheading] OR "surgery"[All Fields] OR "surgical procedures, operative"[MeSH Terms] OR ("surgical"[All Fields] AND "procedures"[All Fields] AND "operative"[All Fields]) OR "operative surgical procedures"[All Fields] OR "general surgery"[MeSH Terms] OR ("general"[All Fields] AND "surgery"[All Fields]) OR "general surgery"[All Fields] OR "surgery s"[All Fields] OR "surgerys"[All Fields] OR "surgeries"[All Fields])))

|  | | |
| --- | --- | --- |
| **2. Embase Search** | Query | Items found |
| #1 | Search 'frailty'/exp OR frailty | 37,373 |
| #2 | Search frail | 31,349 |
| #3 | Search #1 OR #2 | 55,025 |
| #4 | Search 'gastric cancer'/exp OR 'gastric cancer' OR (gastric AND ('cancer'/exp OR cancer)) | 209,095 |
| #5 | Search 'stomach cancer'/exp OR 'stomach cancer' OR (('stomach'/exp OR stomach) AND ('cancer'/exp OR cancer)) | 229,476 |
| #6 | Search 'gastrectomy'/exp OR gastrectomy | 79,951 |
| #7 | Search 'gastric surgery'/exp OR 'gastric surgery' OR (gastric AND ('surgery'/exp OR surgery)) | 250,242 |
| #8 | Search #4 OR #5 OR #6 OR #7 | 475 |
| #9 | Search #9 AND ('Article'/it OR 'Article in Press'/it OR 'Letter'/it) | 224 |
|  |  |  |

| **3.** **Web of Science** | Query | Items found |
| --- | --- | --- |
| #1 | Search ALL = (“gastric cancer” OR “stomach cancer” AND “gastrectomy” OR “gastric surgery”) | 21,372 |
| #2 | Search “Frailty” OR “Frail" (Search within all fields of #1) | 50 |
|  |  |  |
|  |  |  |
|  |  |  |
| **4. Cochrane Library** | Query | Items found |
| #1 | Search All Text: **(frailty) OR (frail)** | 4,757 |
| #2 | Search All Text: **(gastric cancer) OR (stomach cancer) OR (gastrectomy) OR (gastric surgery)** | 16,715 |
| #3 | Search #1 AND #2 | 28 |

Additionally, a manual search was performed in the reference lists of pertinent articles.
