# Supplementary material for: Impact of frailty on survival and readmission in patients with gastric cancer undergoing gastrectomy: A meta-analysis
Source: Front Oncol. 2022 Oct 31;12:972287. doi: 10.3389/fonc.2022.972287 (PMC9659614; doi:10.3389/fonc.2022.972287)
Supplement: Supplementary file 2 [file Table_1.docx]

Supplemental Table S1 Quality assessment of the included studies.

| Author/Year | Representativeness of the exposed cohort | Selection of the non exposed cohort | Ascertainment of exposure | Demonstration that outcome was not present at study start | Comparability of cohorts based on the design or analysis | Assessment of outcome | Enough follow-up periods (>1 year) | Adequacy of follow-up of cohorts | Total NOS |
| --- | --- | --- | --- | --- | --- | --- | --- | --- | --- |
| Tegels 2014 (18) | ★ | ★ | ★ | ★ | ★★ | ★ |  | ★ | 8 |
| Choe 2017 (12) | ★ | ★ | ★ | ★ | ★ | ★ |  | ★ | 7 |
| Lu 2017 (13) | ★ | ★ | ★ | ★ | ★ | ★ | ★ | ★ | 8 |
| Tanaka 2019 (15) |  | ★ | ★ | ★ | ★ | ★ | ★ | ★ | 7 |
| Misawa 2020 (16) |  | ★ | ★ | ★ | ★★ | ★ | ★ | ★ | 8 |
| Kim 2020 (17) | ★ | ★ | ★ | ★ | ★★ | ★ |  | ★ | 8 |
| Osaki 2021 (24) | ★ | ★ | ★ | ★ | ★ | ★ |  | ★ | 7 |
| Jeong 2022 (23) | ★ | ★ | ★ | ★ | ★ | ★ | ★ | ★ | 8 |
| Lee 2022 (25) | ★ | ★ | ★ | ★ | ★★ | ★ |  | ★ | 8 |

NOS, Newcastle-Ottawa Scale.
