# Supplementary material for: Impact of frailty on survival and readmission in patients with gastric cancer undergoing gastrectomy: A meta-analysis
Source: Front Oncol. 2022 Oct 31;12:972287. doi: 10.3389/fonc.2022.972287 (PMC9659614; doi:10.3389/fonc.2022.972287)
Supplement: Supplementary file 3 [file Table_2.docx]

Table S2 GRADE of evidence of outcomes in the included studies

| Outcomes |  | Certainty assessment | | | | | Effect | | Certainty | Importance |
| --- | --- | --- | --- | --- | --- | --- | --- | --- | --- | --- |
|  | No. of study | Risk of bias | Inconsistency | Indirectness | Imprecision | Publication bias | No. of patients | RR (95% CI) |  |  |
| Overall survival | 6 | Not serious | Not serious | Not serious | Not serious | Not serious | 2045 | RR1.94 (1.55 to 2.42) | ⊕⊕⊕⊕ **High** | Critical |
| DFS | 3 | Not serious | Not serious | Not serious | Serious | Unclear# | 492 | RR 1.94 (1.34 to 2.83) | ⊕⊕⊝⊝ **Low^** | Critical |
| Readmission | 2 | Not serious | Not serious | Not serious | Serious | Unclear# | 749 | RR 3.63 (1.87 to 7.06) | ⊕⊕⊝⊝ **Low^** | Critical |

CI, confidence interval; RR, risk ratio. DFS, disease-specific survival; Risk of bias; Serious, there was study with Newcastle-Ottawa Scale <7; Inconsistency: Serious, I ^2^ > 50%; Indirectness of evidence, no indirectness of evidence was found in any study. Imprecision (based on sample size): Serious, n < 1000 participants. # Begg’s test and Egger’s test are not run to evaluate publication bias due to the small number of studies included.

^ Downgraded by one level for imprecision (small number of participants) and downgraded by one level for unclear risk of publication bias.

**GRADE criteria**

**High quality:** Further research is very unlikely to change our confidence in the estimate of effect.
**Moderate quality:** Further research is likely to have an important impact on our confidence in the estimate of effect and may change the estimate.
**Low quality:** Further research is very likely to have an important impact on our confidence in the estimate of effect and is likely to change the estimate.
**Very low quality:** We are very uncertain about the estimate.
